# Nontuberculous Mycobacteria Persistence in a Cell Model Mimicking Alveolar Macrophages

**DOI:** 10.3390/microorganisms7050113

**Published:** 2019-04-26

**Authors:** Sara Sousa, Vítor Borges, Ines Joao, João Paulo Gomes, Luisa Jordao

**Affiliations:** 1 Instituto Nacional de Saúde Doutor Ricardo Jorge (INSA), Departamento de Saúde Ambiental (DSA), Unidade de Investigação e Desenvolvimento (UID), Avenida Padre Cruz, 1649-016 Lisboa, Portugal; ssousa91@gmail.com (S.S.); maria.jordao@insa.min-saude.pt (L.J.); 2 Departamento de Doenças Infeciosas, Instituto Nacional de Saúde Doutor Ricardo Jorge, Núcleo de Bioinformática, Avenida Padre Cruz, 1649-016 Lisboa, Portugal; vitor.borges@insa.min-saude.pt (V.B.); J.Paulo.Gomes@insa.min-saude.pt (J.P.G.); 3 Laboratório Nacional de Referência de Micobactérias, Departamento de Doenças Infeciosas, Instituto Nacional de Saúde Doutor Ricardo Jorge, Avenida Padre Cruz, 1649-016 Lisboa, Portugal; ines.joao@insa.min-saude.pt

**Keywords:** Nontuberculous Mycobacteria (NTM), host-pathogen interaction, macrophages, pH, virulence

## Abstract

Nontuberculous Mycobacteria (NTM) respiratory infections have been gradually increasing. Here, THP-1 cells were used as a model to evaluate intracellular persistence of three NTM species (reference and clinical strains) in human alveolar macrophages. The contribution of phagosome acidification, nitric oxide (NO) production and cell dead on NTM intracellular fate was assessed. In addition, strains were characterized regarding their repertoire of virulence factors by whole-genome sequencing. NTM experienced different intracellular fates: *M. smegmatis* and *M. fortuitum* ATCC 6841 were cleared within 24h. In contrast, *M. avium* strains (reference/clinical) and *M. fortuitum* clinical strain were able to replicate. Despite this fact, unexpectedly high percentages of acidified phagosomes were found harbouring rab7, but not CD63. All NTM were able to survive *in vitro* at acidic pHs, with the exception of *M. smegmatis*. Our data further suggested a minor role for NO in intracellular persistence and that apoptosis mediated by caspase 8 and 3/7, but not necrosis, is triggered during NTM infection. Insights regarding the bacteria genomic backbone corroborated the virulence potential of *M. avium* and *M. fortuitum*. In conclusion, the phenotypic traits detected contrast with those described for *M. tuberculosis*, pointing out that NTM adopt distinct strategies to manipulate the host immune defense and persist intracellularly.

## 1. Introduction

Nontuberculous Mycobacteria (NTM) are a heterogeneous group formed by more than 180 species (http://www.bacterio.net/mycobacterium.html) of environmental microorganisms with distinct human pathogenesis and geographic distribution. Although being mostly saprophytic, NTM can cause opportunistic infections mainly in immunosuppressed individuals. Unlike tuberculosis, reports of NTM infections, which are following a rising trend since acquired immune deficiency syndrome (AIDS) pandemic, are not mandatory, thereby hampering accurate knowledge of its impact on public health. NTM can cause lymphadenitis, disseminated infection, skin and soft tissue infections, but the most common clinical manifestation is pulmonary infection, particularly in immunosuppressed patients and patients with previous pulmonary diseases [1,2,3]. The clinical symptoms of pulmonary infection by NTM are similar to other pulmonary diseases, which makes the accurate diagnosis more difficult [2,4]. The lack of knowledge on NTM is still an issue, with inaccurate diagnosis contributing to disease progression and the deterioration of the patients’ health.

Because NTM are hydrophobic bacilli, they are easily aerosolized [5]. In pulmonary infections, the primary contact, after the inoculation, is the alveolar macrophage, which phagocyte mycobacteria and promote different defense mechanisms, culminating in the cell and mycobacteria death in immunocompetent patients [4,6]. NTM are resistant to most of the available antibiotics and antimicrobials [3,7] being of key importance understanding the human-mycobacteria interactions, and how NTM are capable to survive the host defense mechanisms. This will contribute to the knowledge of the NTM pathophysiology, and may contribute to the development of new antimicrobials.

The ability developed by facultative intracellular pathogens to avoid the host immune system plays a key role in infection outcome. *Mycobacterium tuberculosis*, one of the most successfully facultative intracellular pathogens, blocks phagolysosome biogenesis [8,9]. This mechanism of innate immunity, assures the destruction of pathogens in the harsh environment of phagolysosome, which has acidic pH, a high enzymatic content (lipases, hydrolases, proteases) and antimicrobial molecules (e.g., defensins) [9]. Induction of oxidative stress response, autophagy, cell death, and translocation to the cytosol are other mechanisms reported to affect mycobacterial survival [10]. Nevertheless, for the heterogeneous group of NTM the knowledge on the host response is still scarce. For this reason, we decided to follow the intracellular fate of clinically relevant rapidly (*M. fortuitum*) and slowly (*M. avium*) growing mycobacteria, as well as the rapidly growing model microorganism (*M. smegmatis*) in a cell model that mimics alveolar macrophages. NTM phagosome maturation, NO production, cell death triggered by necrosis and apoptosis were investigated in order to understand if they play a role in intracellular fate of these microorganisms. Since it is known that both host and pathogen play a role in mycobacterial infection outcome, special attention was also payed to NTM. Whole genome sequencing of the studied NTM was performed in order to search for genes that could account for differential virulence features.

## 2. Materials and Methods

### 2.1. Mycobacterial Strains

*Mycobacterium smegmatis* mc^2^155, *Mycobacterium fortuitum* ATCC6841, *Mycobacterium avium* ATCC25921, one clinical isolate of *M. fortuitum* (747/08) and another of *M. avium* (60/08) were grown in Middlebrook’s 7H9 broth medium (Difco, Becton, Dickinson & Company, Sparks, MD, USA) supplemented with 10% Oleic Albumin Dextrose Catalase supplement (OADC, v/v; Difco) and 0.05% Tween 80 (v/v; Sigma, St. Louis, MO, USA) until exponential phase at 37°C/5% CO_2_. The clinical strains were identified as previously described by Joao and colleagues [11] and the antibiotic susceptibility of *M. fortuitum* 747/08 (Clarithromycin: Intermediate; Doxycycline: Resistant; Amikacin and sulfamethoxazole: Susceptible) and *M. avium* 60/08 (Clarithromycin: Susceptible) was determined following Clinical & Laboratory Standards Institute (CLSI) guidelines [12].

### 2.2. THP-1 Cells

THP1 cells (ATCC TIB-202) were grown in RPMI 1640 (Lonza, Basel, Switzerland) supplemented with 10% fetal bovine serum (FBS; Lonza), 2 mM L-glutamine (Gibco, Life Technologies Corporation, Grand Island, NY, USA), 10 mM Hepes (Gibco), 1 mM sodium pyruvate (Gibco), 4500 mg/ L glucose (Gibco), and 50 μg/mL gentamicin (Gibco) at 37 °C/5% CO_2_. The cells were seeded onto 96-well culture dishes at a density of 4 × 10^4^ cells per well and treated for 72 h with 100 nM phorbol myristate acetate (PMA-Sigma). Cells were washed thrice with PBS and further incubated for 24 h in cell culture media without PMA. These differentiated cells will be referred to as macrophages.

### 2.3. Macrophage Infection

Single cell suspensions of mycobacteria in cell culture media without antibiotics (infection media) with an OD_600nm_ = 0.1 were prepared from bacterial cultures in exponential phase and used for macrophage infection as previously described [13]. Macrophages were allowed to uptake rapidly growing mycobacteria (RGM) for 1h and slowly growing mycobacteria (SGM) for 3 h. At infection time (1 or 3 h), and after several hours (4, 8, 24 h for RGM) or days (1, 3, 5 and 7 days for SGM) infected macrophages were washed with PBS and lysed with an aqueous solution of 1% igepal (v/v; Sigma). Serial dilutions of the lysate were prepared in water and plated on Mueller-Hinton agar (RGM) or Middelbrook 7H10 supplemented with 10% OADC (SGM). After approximately 3 or 10 days of incubation at 37 °C, colony-forming units (CFU) were enumerated for RGM or SGM, respectively. The selected time points for analysis took into account the generation time of the different mycobacteria, following a previously applied protocol [13].

### 2.4. Phagosome Maturation 

Macrophages grown on glass cover slips in 24-wells cell culture plates were infected with Oregon green (Molecular Probes, Invitrogen, Eugene, OR, USA) stained *M. avium* or *M. fortuitum* strains in the conditions described under 2.3. 

Lysotracker Red DND-99 (Molecular Probes) staining of acidic organelles was carried out by adding a 1:5000 dilution in infection media that was added for the last 30 min of the experiments. Cells were washed thrice with PBS, fixed with 4% paraformaldehyde in PBS for 15 min at room temperature (RT) in the dark, washed with PBS and mounted on a glass slide by inverting the coverslip on a drop of mounting medium (Dako, Lisbon, Portugal). Glass slides were stored, protected from light, at 4 °C until observation under a Leica SP2 confocal microscope.

For immunofluorescence, after fixation cells were permeabilized with 0.1% Triton X-100 (Sigma) for 5 min at RT, washed twice with PBS and further incubated in 3% bovine serum albumin fraction V (Merck, Lisbon, Portugal) in PBS (blocking solution) for 20 min. Cells were then incubated with 5 μg/mL primary antibody (mouse anti-human CD63 from Pelicluster) in blocking solution (20 min, 37 °C in a humidified atmosphere protected from light), washed twice with PBS and further incubated with a 1:1000 dilution of secondary antibody anti-mouse Alexa 647 (Molecular Probes) in blocking solution in the same conditions. Macrophages were washed with PBS and the procedure was repeated using as primary antibody rabbit anti-human rab7 (Santa Cruz Biotechnology Inc., Dallas, TX, USA) and secondary antibody anti-rabbit Alexa548 (Molecular Probes). Macrophages were washed and further processed for confocal analysis, as described for Lysotracker Red. 

Only phagosomes containing single NTM were counted. Phagosomes containing NTM clumps were not considered for analysis purposes. At least 100 phagosomes containing only one NTM were scored. At least three independent experiments were performed.

### 2.5. NTM Growth at Different PHs

NTM growth was evaluated at pH 6.7; 5.4 and 4.6 using a Bactec MGIT960 apparatus (Becton, Dickinson and Company, Sparks, MD, USA) for 45 days following the manufacturer’s instructions with small modifications. Briefly, the Mycobacteria Growth Indicator Tube (MGIT)(media supplemented with 10% (OADC) was adjusted to the desirable pH by adding 1M HCl (Merck) and was inoculated with 10^4^ CFU/mL. MGIT media at different pHs were used as sterility controls. The assay was performed in duplicate and the fluorescence readings were recorded daily.

### 2.6. Nitric Oxide

Nitrite concentration indicating the NO production was evaluated in supernatants of macrophages infected with NTM using the standard Griess reaction adapted to microplate, as previously described [13]. The detection and quantification limits of the method were 1.8 and 9 μM NO, respectively. Supernatant of uninfected cells was used as negative control and supernatant of cells treated for 20h with 100 IU human γ-IFN (Immunotools, Friesoythe, Germany) and 20 ng/mL *K. pneumoniae* lipopolysaccharide (LPS—Sigma) was used as positive control.

### 2.7. Apoptosis Mediated by Caspase 8 and Caspases 3/7

Apoptosis induced by clinical strains of *M. avium* and *M. fortuitum* mediated by caspase 8 and caspase 3/7 was evaluated after 0.25; 0.5, 1, 2, 3 and 4 h or 1, 2, 3, 6 and 24h, respectively, using the carboxyfluorescein FLICA kit (Pharmigen by BD Biosciences, Oeiras, Portugal) according to the manufacturer’s instructions. For both assays, the cells were seeded in 24-well plates at a density of 5x10^4^ cells per well, on circular glass coverslips. After 1h of incubation with carboxyfluorescein FLICA, cells were washed with PBS and fixed with 4% PFA for 15minutes at RT in the dark. Cells were then mounted by inversion on a drop of fluorescence mounting medium previously placed on a glass slide with 1μL of Hoechst dye for nuclear staining supplied in the kit. The samples were stored at 4 °C, protected from light, until visualization through confocal microscopy (Leica, SP2) at a magnification of 400×. At least two independent experiments were performed in duplicate. For positive control, apoptosis was induced by incubating cells with 1 μg/mL of LPS from *K. pneumoniae* during 24h and 3 mM of ATP (Sigma) for 30 min. Uninfected cells were used as negative control.

### 2.8. Statistical Analysis

Results of at least three independent experiments were expressed as the means +/− standard deviation. Statistical significance was assessed by the Student t-test two-tailed. A *p* < 0.05 was considered statistically significant.

### 2.9. Whole-Genome Sequencing, Assembly and Annotation

Mycobacteria were grown on 7H10 Middlebrook agar supplemented with 10% OADC. DNA was extracted using phenol chloroform method [14], quantified through a fluorometric method (Qubit assay, Quant-iT double-stranded DNA assay kit, broad range by Life Technologies, Carlsbad, CA, USA) and subsequently subjected to the Nextera XT library preparation protocol (Illumina Inc., San Diego, CA, USA) prior to paired-end sequencing (2 × 250 bp) on a MiSeq equipment (Illumina), according to the manufacturer’s instructions. 

Genome sequences were assembled using INNUca v3.1 (https://github.com/B-UMMI/INNUca), which is an integrative bioinformatics pipeline that involves: (i) reads’ quality analysis (FastQC v0.11.5; http://www.bioinformatics.babraham.ac.uk/projects/fastqc/) and cleaning/improvement (Trimmomatic v0.36) [15]; (ii) de novo genome assembly (SPAdes v3.10) [16] and post-assembly optimization using Bowtie2 v2.2.9 [17], samtools v1.3.1 [18], and Pilon v1.18 [19]; and (iii) QA/QC (such as depth of coverage and number of contigs) reporting throughout the analysis. Final polished assemblies were annotated with Prokka v1.12 [20]. The assembled contigs (.fasta and .gbk files), the nucleotide sequences of the prediction transcripts (CDS, rRNA, tRNA, tmRNA, misc_RNA) (.ffn files) and the respective amino acid sequences of the translated CDS sequences (.faa files) are available at http://doi.org/10.5281/zenodo.2384513. All raw sequence reads used in the present study were deposited in the European Nucleotide Archive (ENA) (BioProject PRJEB30455). Detailed ENA accession numbers, as well as the draft genome length, mean depth of coverage, number of contigs and predicted CDS are described in Table S1.

### 2.10. Overview of the Accessory Genome of Same-Species Reference and Clinical Isolates

In order to search for genomic regions that are differentially present in the same-species reference and clinical isolates, chewBBACA v2.0.11 suite (https://github.com/B-UMMI/chewBBACA) [21] was applied to create a pan-genome loci panel (CreateSchema module; default settings) for each species (*M. avium* and *M. fortuitum*), using the two assemblies of the representative strains (reference and clinic). Subsequently, draft genome sequences and CDSs of each strain were queried against the respective species panel using chewBBACA AlleleCall module (default settings) to identify “accessory genome” gene clusters present in one strain that have no homologous hits in the other same-species strain (using a BLAST Score Ratio—BSR—threshold of 0.6). In a conservative manner, small gene clusters (< 5Kbp) and potential strain-specific genes outside clusters were not extracted to avoid false positives due to differential contigs fragmentation. All species-specific chewBBACA runs included training files generated by Prodigal v2.6.3 from the following reference genomes: *M. avium* 104 (RefSeq Accession CP000479.1) and the *M. fortuitum* CT6 (NZ_CP011269.1). Draft-genome alignments were also constructed using the progressive algorithm of Mauve software (version 2.3.1) (http://darlinglab.org/mauve/mauve.html) to visually inspect the detected regions and check positional orthologues. Clusters of Orthologous Groups (COGs) categories were assigned to the detected proteins using “cdd2cog” script [22] after RPS-BLAST+ (Reverse Position-Specific BLAST) (e-value cut-off of 1e-2), where only the best hit (lowest e-value) and first COG were considered.

### 2.11. In silico Screening of Virulence and Antibiotic Resistance Genes 

In order to screen the genomes of the five NTM strains for the presence/absence of known virulence factors, a custom sequence database was created based on a comprehensive list of genes recently gathered by Fedrizzi and colleagues [23], as well as, additional virulence factors retrieved from literature [24,25,26,27,28,29,30,31,32] or from the Virulence Factor Database (VFDB) [33]. A detailed description of the list of searched virulence-associated genes, namely the “locus_tags” in the reference genomes from which individual CDS sequences were extracted, is presented in Table S2. This in-house sequence database was applied to query both draft genome sequences and CDSs (inferred using prokka) with the blastp BSR-based approach implemented in the chewBBACA v2.0.11 suite [21], using a BSR threshold of 0.5 and a training file generated by Prodigal v2.6.3 from the H37Rv reference genome (RefSeq Accession NC_000962.3). The BLASTn-based ABRIcate v0.8 tool (https://github.com/tseemann/abricate) was also applied to query the assemblies against the custom virulence factors database, as well as against the following databases of AMR-associated markers: ResFinder, NCBI, ARG-ANNOT and CARD (versions released with Abricate v.08, March 2018). 

Finally, we also sought to infer the subspecies of the NTM strains by performing additional BLASTn comparative analyses using previously described markers for species/subspecies discrimination, namely the traditional 16S rRNA and hsp65 genes [11,34], the IS1311 (GenBank accession no. U16276), IS900 (GenBank accession no. X16293), IS901 (GenBank accession no. X59272) and DT1 (GenBank accession no. L04543) markers for *M. avium* [35], and dnaK gene (GenBank accession no. HQ259919 and GU362437) for *M. fortuitum* [36]. *M. avium* 60/08 and *M. fortuitum* 747/08 could be classified as *M. avium subspecies hominissuis* and *M. fortuitum subspecies acetamidolyticum*, respectively.

## 3. Results

### 3.1. Kinetics of NTM Survival in Macrophages

**Figure 1:**
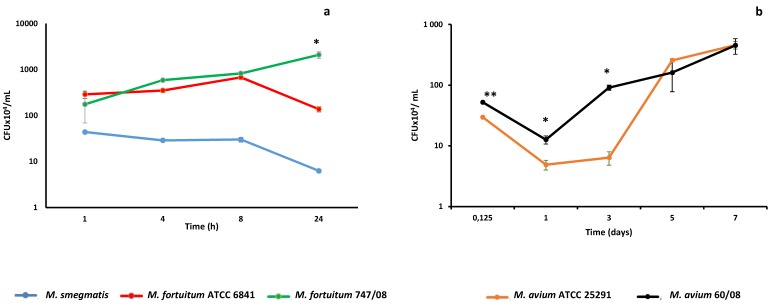
Nontuberculous mycobacteria (NTM) persistence within macrophages. Intracellular persistence of rapidly growing mycobacteria (**a**) and slowly growing mycobacteria (**b**) was followed over 1- and 7-day periods, respectively. (* *p* < 0.05; ** *p* < 0.01).

From our previous work, we knew that human monocyte derived macrophages (HMDM) clear the saprophyte mycobacteria *M. smegmatis* within 24–48h [13]. Here we followed *M. smegmatis* survival kinetics in THP-1 derived macrophages that mimic alveolar macrophages [37], a cell that plays a crucial role in mycobacteria lung infections [38]. As shown in Figure 1a, macrophages were able to clear *M. smegmatis* within 24h. Next, we challenged macrophages with reference and clinical strains from *M. fortuitum* and *M. avium*, which are two of the most clinically relevant RGM and SGM species, respectively [39,40]. Although no significant difference in the uptake of the two *M. fortuitum* strains by macrophages was observed (*p* = 0.318), the survival kinetics followed different trends, with the *M. fortuitum* ATCC 6841 following a kinetic that resembles the one observed previously for *M. smegmatis* in J774 macrophages [41]. *M. fortuitum* ATCC 6841 is able to persist and even replicate up to eight hours, but is cleared by macrophages after 24h (Figure 1a). Despite both the reference and clinical strains of *M. fortuitum* exhibit similar kinetics until 8 h, after this point *M. fortuitum* 747/08 replicated between 8h and 24h, reaching a significantly higher number of CFU than the reference strain (*p* = 0.0023) (Figure 1a). This duality in kinetic profiles was not observed for *M. avium* since both reference (ATCC 25291) and clinical (60/08) strains (Figure 1b) were able to replicate within macrophages. Of note, from infection time (3h) until 3 days the number of CFU was significantly higher for *M. avium* 60/08 than for *M. avium* ATCC 25921 (*p* < 0.05). Afterward, no statistically significant difference was observed between CFU counts for the two *M. avium* strains. 

### 3.2. NTM Phagosome Maturation and pH

**Figure 2:**
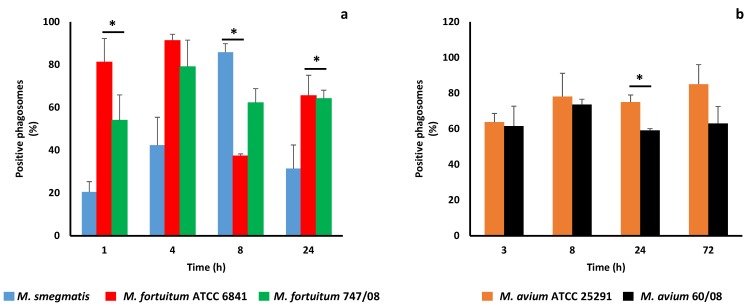
Acidification of phagosomes containing nontuberculous mycobacteria. Acquisition of Lysotracker by rapidly growing mycobacteria (**a**) and slowly growing mycobacteria (**b**) was used to assess phagosome acidification. (* *p* < 0.05).

Phagosome maturation culminates with phagolysosome formation, a crucial organelle for both cell housekeeping and pathogen elimination [42]. It has been known for a long time that pathogenic mycobacteria, namely *M. tuberculosis* [8] and *M. avium* [43] block phagosome maturation at an early stage avoiding phagolysosome formation and exposure to its killing machinery. A characteristic feature of this organelle is its acidic pH ranging between 4.5 and 5.5 [44]. Here we started by evaluating the pH of mycobacterial phagosomes using Lysotracker. The results are shown in Figure 2a,b for RGM and SGM, respectively.

As soon as 4h infection all RGM, except *M. smegmatis*, revealed more than 40% of Lysotracker positive phagosomes (acidic phagosomes). For *M. avium*, a higher percentage of acidified phagosomes (60%), assessed by Lysotracker acquisition was reached after 3h (Figure 2b). These results are surprising since not only the percentage of acidic phagosomes is higher than expected, but also because the lowest percentage of acidic phagosomes (i.e., higher efficiency in blocking phagosome maturation) belongs to the saprophyte mycobacteria (*M. smegmatis*), which is more quickly killed by macrophages (Figure 1a). For the two strains of *M. fortuitum* no statistically significant difference between the percentage of acidic phagosomes was found over time, with the lowest value being registered at 8h for the reference strain (Figure 2a). For *M. avium*, a significant difference between reference and clinical strain was observed at 24h, when *M. avium* 60/08 registered the lowest percentage of acidic phagosomes (59%, Figure 2b). 

**Figure 3:**
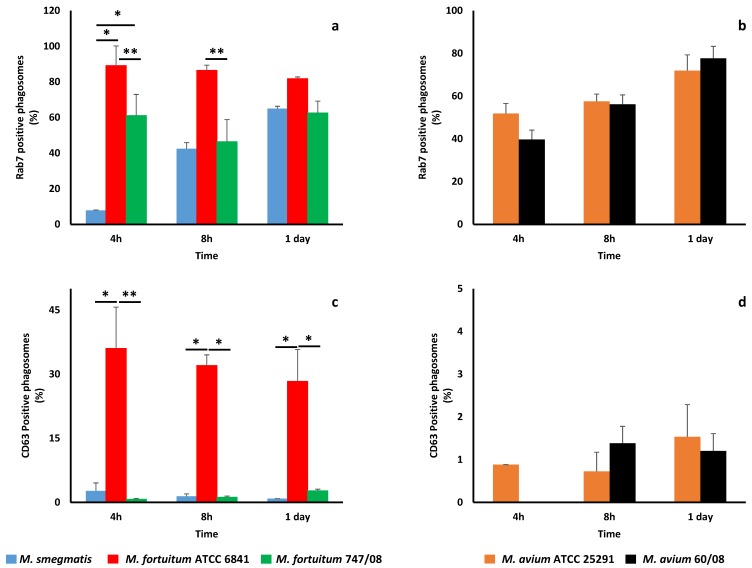
Maturation of phagosomes containing nontuberculous mycobacteria. The acquisition of a late phagosome marker rab7 by phagosomes containing *M. smegmatis*, *M. fortuitum* ATCC 6841 and 747/08 (**a**), as well as, *M. avium* ATCC 25291 and 60/08 (**b**) were evaluated at 4, 8 and 24 h. Acquisition of the lysosome marker CD63 by phagosomal membrane of rapidly (**c**) and slowly (**d**) growing mycobacteria phagosomes were also monitored. (*p < 0.05; ** *p* < 0.01).

In order to confirm if the studied NTM were unable to prevent phagosome maturation, we looked at rab7 (late phagosome marker) and CD63 (lysosome marker) acquisition by mycobacterial phagosomes. All NTM phagosomes acquired rab7 (Figure 3a,b), although following different kinetics. As observed with Lysotracker acquisition, *M. smegmatis* was the bacterium that acquired less rab7 at early stages (4 h). Furthermore, this difference in rab7 acquisition by *M. smegmatis* was statistically significant when compared to both *M fortuitum* strains (*p* < 0.05). *M. fortuitum* ATCC6841 phagosomes acquired more rab7 at all-time points, with significant difference from *M. fortuitum* 747/08 at 4 and 8 h (Figure 3a). These results are in good agreement with phagosome acidification monitored by Lysotracker acquisition. For *M. avium*, no significant difference in rab7 acquisition by phagosomes was observed between the reference and clinical strains at all time points (Figure 3b).

Regarding CD63, this lysosome marker was acquired in less amounts than rab7 by RGM (Figure 3c) and SGM (Figure 3d) phagosomes. *M. fortuitum* ATCC 6841 was the bacterium that more efficiently acquired CD63, or in other words, was less effective in blocking phagosome maturation.

Finally, we evaluated the ability of NTM to persist in vitro at pHs that mimic those found in different compartments of the phagocytic/endocytic pathway. In macrophages the luminal pH of early phagosomes/endosomes is around 6.2, while in late phagosomes/ endosomes is around 5.5 and in lysosomes is 4.5–5.0 [9,45]. *M. fortuitum* and *M. avium* strains were able to replicate at all pHs in vitro whereas *M. smegmatis*, although being able to replicate at mild acidic pHs (5.4 and 6.7), was killed at pH 4.6 (Table 1). 

### 3.3. Nitric Oxide

The role of reactive nitrogen intermediates (RNI) including nitric oxide (NO) in mycobacterial pathogenesis is far from consensus and likely depends on multiple factors (such as host- or mycobacteria-specific factors) [13,41,46,47,48,49,50,51]. Since the maximum microbicidal activity of NO is achieved at acidic pH and NTM reached unexpected levels of phagosome acidification, we decided to evaluate the content of this mediator in supernatants of macrophages infected with NTM. The levels of NO were residual for all NTM during infection time course. NO was only found in quantifiable concentrations at day 7 for *M. avium* 60/08 strain. Furthermore, NO concentration in infected activated macrophages (22.89 µM) was higher than in resting macrophages (13.59 µM) suggesting that NO might play a minor (or no) role in NTM persistence within human macrophages. This result is in good agreement with our previous findings for *M. smegmatis* (unpublished data) and other mycobacteria [13] as well as with other published studies [52,53,54,55,56].

### 3.4. Apotosis

**Figure 4:**
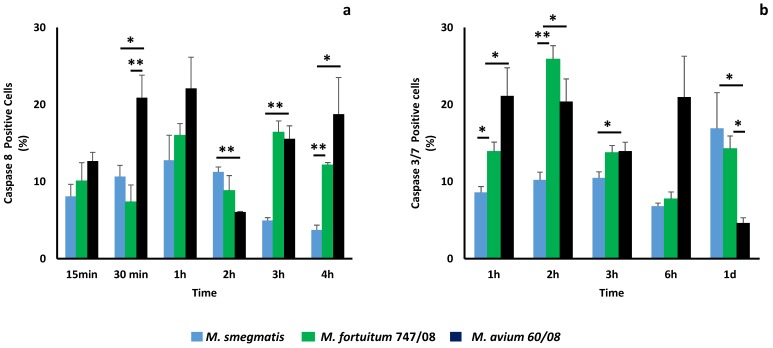
Apoptosis induction during nontuberculous mycobacteria infection. Apoptosis induction mediated by caspase 8 (**a**) and caspase 3/7 (**b**) in macrophages infected with *M. smegmatis*, *M. fortuitum* 747/08 or *M. avium* 60/08 was evaluated. (* *p* < 0.05; ** *p* < 0.01).

In order to investigate the role played by cell death in the different intracellular persistence profiles observed in this study, we selected *M. smegmatis* (which is killed by macrophages), *M. fortuitum* 747/08 and *M. avium* 60/08 (both persist within the macrophages) for a more detailed study. Necrosis was monitored by propidium iodide acquisition, being residual (less than 3%) for all NTM at all-time points (data not shown). Programmed cell death induction, mediated by caspases 8 (activator), 3 and 7 (effectors) was followed (Figure 4). Apoptosis is triggered as soon as 15 min after infection with these NTM, as shown for caspase 8 in Figure 4a. *M. fortuitum* 747/08 and *M. avium* 60/08 are more effective apoptosis inducers than *M. smegmatis*, with this difference statistically significant at 30 min, 3 h and 4 h. Curiously, at 2h, the opposite result is observed (Figure 4a). For the SGM (*M. avium* 60/08) the induction of caspase 8 for additional time points (8, 24 and 72 h) was evaluated. The results obtained at 8, 24 and 72 h were 13.9% ± 0.33%, 4.24% ± 0.59% and 9.37% ± 1.43% (data not shown), respectively, which allowed us to conclude that caspase 8 activation peaked after 1h of infection. The results obtained for caspase 3/7 confirm the observed trend for caspase 8 with *M. fortuitum* 747/08 and *M. avium* 60/08 being stronger apoptosis inducers until 6h (Figure 4b). After 1 day, the SGM (*M. avium* 60/08) induced significantly less apoptosis mediated by caspase 3/7 than the RGM (*M. smegmatis* and *M. fortuitum* 747/08).

### 3.5. Main Genetic Differences between Same-Species Reference and Clinical Isolates

Whole-genome sequencing (WGS) of the five NTM strains revealed genome lengths and CDS counts ranging from ~4.9Mbp (4570 predicted CDSs) for *M. avium* reference strain (ATCC25291) to ~6.9Mbp (6713 CDSs) for the *M. fortuitum* clinical strains 747/08 (Table S1). All genome statistics fit what it is described for each species in the NCBI genome database (raw NGS data and derived genome and predicted CDS/protein sequences generated in this study are publicly available (see Table S1 and http://doi.org/10.5281/zenodo.2384513). Of note, the clinical isolates presented estimated genome lengths about 0.55 Mbp larger than their counterpart reference strains (Table S1). In this context, although distinct phenotypic signatures among same-species strains can rely on a multitude of genetic differences (from discreet SNPs to extended differences in gene content), we sought to get a global picture on the main accessory genome differences between the same-species reference and clinical strains (results are detailed in Table S3). Not surprisingly, most of the differentially present genes have unknown or poorly characterized functions. It is still noteworthy that, discounting these, the most frequently detected category was “Lipid transport and metabolism”. 

### 3.6. Repertoire of Virulence and Antibiotic Resistance Genes

The host and the pathogen determine the outcome of mycobacteria infection. In this context, after studying the role played by several innate immunity mechanisms in the cell model, NTM genomes were characterized for the presence/absence of virulence factors by screening a custom database constructed based on list of curated genes gathered from literature (see details in Table S2). Main gene classes playing a pivotal role in mycobacteria virulence such as ESX export systems, PE/PPE proteins, Mce proteins, Sec-dependent secretion system and Tat export system were included [23]. *Mycobacterium* protein secretion via ESX export systems (also called Type VII secretion systems) are involved in important virulence mechanisms, including macrophages escape [57]. In total, mycobacteria can encode up to five ESX systems (ESX1 to 5) with differential functions [57]. Here, the ESX-3 system, which can be important for in vitro growth due to its involvement in iron and zinc fixation [58] was found to be encoded by all NTM (Table S2). Still, it is worth noting that the ESX-3 gene coding for PPE4, which shares the same operon as PE5 (a potential modulator of innate immunity and survival in macrophages) in *M. tuberculosis*, was found to be absent in all studied genomes. ESX-2 and ESX-5 are associated specifically with SGM [59]. In good agreement to the literature, genes coding for the ESX-2 system were exclusively found in *M. avium* strains. The same trend was observed for ESX-5, with exception of cyp143 gene, for which an orthologue gene was detected in the RGM *M. fortuitum* 747/08. By contrast, genes encoding LipY, PPE41 and PE25 were absent in all studied NTM. Of note, the latter virulence genes, in particular LipY, which likely play a role in the ability of *M. tuberculosis* to compromise immune responses [60], are also rare in most NTM species [23]. With exception of the virulence regulator and nucleoid-associated protein EspR (found to have homologous genes in the five NTM species) [61], all surveyed genes of the ESX-1 system, which together with ESX-5 are two ESX systems mostly associated with virulence [57], were absent in the two *M. avium* strains. Still, ESX-1 genes revealed matches in the other two NTM species. These genes were intriguingly shared by *M. smegmatis* and the *M. fortuitum* ATCC 6841, but not *M. fortuitum* 747/08 (Tables S2 and S3). For instance, this was observed for eccA1-eccB1-eccCa1-eccC1 gene cluster and the genes coding for CFP10/ESAT6 complex. Interestingly, the later complex is believed to act as a regulator of macrophage cell death at different stages of tuberculosis infection [62]. The ESX-4 is the less documented system. We highlight two genes coding for ESAT-6-like proteins related to conjugation (the co-transcribed esxT and esxU), which revealed homologous genes in all studied NTM (concordantly with their finding in most NTM species) [23]. Interestingly, the esxUT transcript, which is activated upon cell-to-cell contact in *M. smegmatis* during conjugation, may function as the primary substrates of the ESX-4-mediated virulence functions [63]. Their presence in most NTM species led us to speculate that these genes play a conserved and key role in triggering the complex interaction-response networks in mycobacteria. 

The Mce (mammalian cell entry) proteins family are closely involved in the invasion and persistence in host cells [64], being present in almost all NTM [23]. Here, we also found homologous proteins for the large majority of Mce proteins in the three NTM species. In an opposite scenario, the majority of genes encoding to PE/PPE proteins, known to be almost exclusive of *M. tuberculosis*, were absent, as expected [23]. 

So as to complement our phenotypic insights in these topics, we also looked at genes likely involved in anti-apoptotic pathways and in the mycobacterial adaptation to acidic environments. First, we search for genes that have been recently linked to the ability of *M. avium* to escape apoptotic macrophages (MAV_2235, MAV_2120, MAV_2410 and MAV_4563) [24]. We found homologous genes in all NTM strains, with exception of MAV_4563 (coding for a protein with unknown function), which is absent in the *M. smegmatis*, and the gene coding for a divalent metal cation transporter (MAV_2120), which was exclusively present in *M. avium* strains. This “*M. avium*-specific” pattern was also found for the gene coding the cysteine synthase A (CysK; MAV_2052), which was recently described as potential mediator of apoptotic cell death through TLR4 dependent ROS production and JNK pathway in murine macrophages [29]. MAV_2054, which is likely involved in inducing macrophage apoptosis by targeting mitochondria, was present in both *M. avium* strains as well as in *M. smegmatis* [30]. Of note, among other conserved virulence factors, we highlight that all five NTM genomes code for: (i) NuoG (NADH-quinone oxidoreductase subunit G), which was implicated in the inhibition of apoptosis in *M. tuberculosis* infected cells [27]; (ii) KatG, which is an antioxidant enzyme with catalase/peroxidase activity that is highly implicated in virulence, namely due to its anti-apoptotic action [32]; and (iii) PknG, a protein kinase G that has been shown to promote mycobacterial survival inside host cells through their potential role as regulator of the mycobacterial growth in an acidic environment [31]. 

Finally, genes responsible for antibiotic resistance were investigated (Table S4), with emphasis on clinical strains, since it might influence bacterial fitness and infection outcome in clinical settings. Macrolide resistance could be linked to erm genes. In fact, Erm(39) homologue gene was found in the intermediately resistant *M. fortuitum* 747/08 but not in the clarithromycin susceptible *M. avium* 60/08, as expected [65]. A similar result was obtained for doxycycline resistance [66], with both tap (associated with an efflux pump) and tet genes (related to antibiotic enzymatic modification) being present in the resistant *M. fortuitum* 747/08.

## 4. Discussion

Here we addressed the fate of saprophyte *M. smegmatis* and two-clinically relevant NTM, an RGM (*M. fortuitum*) and an SGM (*M. avium*) in a cell model that mimics alveolar macrophages. The outcome of mycobacterial infections is determined by multiple host (e.g., genetic and/or immunological factors) and mycobacteria factors (e.g., the arsenal of virulence genes). In this context, we focused on studying several mechanisms of innate immunity response (e.g., phagosome acidification, NO production, cell death) triggered by NTM; as well as, on characterizing these potential pathogens at genomic level by WGS. 

Intracellular fate of the five NTMs was distinct. The saprophyte *M. smegmatis* was rapidly cleared by macrophages. Clinical strains of *M. fortuitum* and *M. avium* were able to persist within macrophages, whereas their reference counterparts were killed to some extent (*M. fortuitum*) or experienced a lag phase before growth started (*M. avium*). We cannot discard that this high success of clinical strains might reflect adaptation to the human host, although one might expect bacterial fitness costs associated with the human infection (especially because NTM are known to be intrinsically resistance to antibiotics). Nevertheless, the resistant *M. fortuitum* 747/08 (resistant to doxycycline and intermediately resistant to clarithromycin) and the susceptible *M. avium* 60/08 (susceptible to clarithromycin) are both able to replicate within macrophages, corroborating that the cost of antibiotic resistance mutations may differ depending on the species/strain genome backbone [67,68]. A similar behavior had been observed for *M. tuberculosis* clinical strains in monocyte-derived macrophages from human peripheral blood [69]. 

In order to understand how mycobacteria are killed by macrophages, we started by monitoring phagosome acidification, since phagolysomes are main players in microorganisms’ elimination and pathogenic mycobacteria are able to block phagosome-lysosome fusion [42,43,70]. This led us to an unexpected result, especially among NTM that are able to persist and replicate within macrophages. Indeed, for all *M. fortuitum* and *M. avium* strains, the percentage of acidic phagosomes assessed by Lysotracker acquisition was always equal or higher than 40% (Figure 2a,b). Surprisingly, *M. smegmatis*, killed by macrophages, started with a modest percentage of acidified phagosomes (20%) that increases up to 80% at 8 h. This result made us questioning the ability of NTM to arrest phagosome maturation and the role played by acidic pH on mycobacteria killing. *M. tuberculosis* phagosome maturation arrest occurs at an early stage between rab5 (early endosome marker) and rab7 (late endosome marker) acquisition [71]. Here, considering the high percentages of acidic phagosomes observed, we hypothesized that NTM phagosome arrest might occur at a later stage, meaning that at least the late endosome marker rab7 was acquired. Our subsequent results confirmed this hypothesis (Figure 3a,b) since rab7 was present in at least 40% of *M. fortuitum* and *M. avim* phagosomes as soon as 4h. *M. smegmatis* was less efficient acquiring the late endosome marker rab7 what is in good agreement with the acidification results (Figure 1a). Next, we followed CD63 acquisition in order to establish if NTM phagosome maturation progresses until phagolysosome formation or is arrested at late phagosome stage. All NTM, acquired residual levels of the phagolysome marker CD63 (less than 2%) except *M. fortuitum* ATCC 6841 that presented at least 30% of positive phagosomes for CD63 (Figure 3c,d). Altogether, our data supports that NTM are less efficient in blocking phagolysome biogenesis since NTM phagosome acquires the late phagosome marker rab7. In other words, our results suggest that NTM are able to inhabit a late phagosome with a theoretical luminal pH around 5.5 [45]. *In vitro*, it has been shown that mycobacteria from *M. tuberculosis* complex are killed until different extents at acidic pH [13,72]. Still, our results suggest that NTM might be more tolerant to lower pHs. In vitro we showed that all NTM were able to persist and replicate in a pH range between 4.4 and 6.7, except *M. smegmatis* that was killed at pH 4.4 (Table 1). This might result from the need of these ubiquitous microorganisms to adapt to harsh environments. Several authors have described the ability of NTM to persist within acidic conditions in soil and water [73,74,75]. Nevertheless, it is known that acidic pH promotes the activity of numerous host defense mechanisms, such as lysosomal hydrolases, ROI and RNI [42,76,77]. Here we focused on RNI, namely in NO production, because this bioactive gas, which is known to play multiple roles in innate and adaptive immune responses, is important to control mycobacteria infection in mouse macrophages [50]. Nevertheless, the role played by RNI and NO in NTM infection, namely *M. avium* is controversial. Dennis and colleagues showed that RNI, but not reactive oxygen intermediates (ROI), are important to control *M. avium* in HMDM [47]; whereas other authors showed that *M. avium* is not susceptible to NO toxic effects in a mouse model [51,78]. In our cell model, NO was detected only for *M. avium* 60/08 after 7 days. As expected, NO production increased in activated macrophages, but as in resting macrophages quantifiable levels were achieved only after 7 days infection with *M avium* 60/08. These results suggest that, in the conditions tested, NO does not play a key role in NTM infection control.

The last mechanism investigated in this work was cell death that, depending on the pathway followed, might play opposite roles in mycobacteria pathogenesis. Apoptosis induction is part of the immune response, facilitating mycobacteria clearance and infection containment [79]. On contrary, necrosis is a virulence mechanism of pathogenic mycobacteria that facilitates infection spread [10,80]. According to this theory, it would be expected that at least *M. avium* 60/08 and *M. fortuitum* 747/08, which would be responsible for human disease, were able to induce necrosis. This was not observed in the conditions tested, with residual levels of necrosis independent of NTM. Furthermore, we observed that *M. avium* 60/08 and *M. fortuitum* 747/08 were significantly stronger inducers of caspase 8 and caspase 3/7 at almost all time points evaluated (Figure 4a,b). An exception was observed for caspase 8 after 2 h infection when *M. smegmatis* was the strongest inducer (Figure 4a). *M. avium* has been the most studied NTM and apoptosis induced by MAP-Kinase activation, TNF-alpha, mitochondria, caspases activation among other pathways have been reported, not only by the whole organism but also by specific components [29,30,81,82,83]. *M. fortuitum* was, just recently considered a relevant human pathogen, which might explain the small number of studies conducted. Nevertheless, the ability of *M. fortuitum* to trigger apoptosis in different hosts by different pathways, namely by caspase activation, and the contribution of cell death in mycobacteria clearance are well documented [48,84,85,86]. Our results suggest that apoptosis mediated by caspase 8 and caspase 3/7 favours *M. fortuitum* and *M. avium* intracellular persistence, at least in the early stages of infection. Although contradictory at first glance, this observation fits previous reports suggesting apoptosis induction as a strategy used by *M. avium* to spread infection [87,88]. Altogether, these results showed that this is not a black-or-white situation and that further studies are needed to elucidate the role played by cell death in NTM pathogenesis. 

The last topic investigated in this study was the bacterium itself, namely its genomic characteristics. As observed by Fredizzi [23], increasing the knowledge on genetic characterization and classification of the NTM species remains a priority, so that novel strategies for the identification at species/ subspecies level of microorganisms in clinical settings could be developed. Here, following the discriminatory criterion described by Shin and colleagues [35], we inferred that *M. avium* 60/08 and *M. avium* ATCC 25921 belong to the *M. avium subspecies hominissuis* and *M. avium subspecies avium*, respectively. For *M. fortuitum*, we confirmed that ATCC 6841 belongs to *M. fortuitum subspecies fortuitum*, and 747/08 would be classified as *M. fortuitum subspecies acetamidolyticum* based on a two nucleotides difference in the dnaK gene [36]. However, as other nucleotide differences were present in this gene, we cannot exclude that this clinical strain would be later on re-classified. This will be definitely disclosed when WGS-based subspecies classification approaches become validated and standardized. 

Considering that we found important phenotypic differences (e.g., intracellular persistence) between reference and clinical strains, we investigated the differential accessory genome between same-species strains (Table S3). As previously observed for group-specific genes in NTM [23], most of the differentially present genes identified here code for proteins with unknown functions. Still, it should be emphasized that proteins involved in “Lipid transport and metabolism” category were among the most exclusively present in clinical strains, regardless of the species, which is noteworthy since the high lipid content is a hallmark of *Mycobacterium* and lipids have been implicated in mycobacteria virulence [89]. Nonetheless, no major differences were found for genes coding for mycolic acids (data not shown), which are among the most important mycobacterial lipids being essential to mycobacteria survival, physiology and fitness [90]. Altogether, these genetic differences between reference and clinical NTM corroborates the assumption that it is challenging to perform genotypic-phenotypic association traits in NTM, where most of the functional diversity remains uncharacterized. Nevertheless, our detailed characterization of the repertoire of known virulence factors found in both reference and clinical strains (including the model microorganism *M. smegmatis*) is a step forward toward improving the current knowledge concerning which genetic traits underly the ability of NTM to cause human disease. This is well illustrated by the detection of multiple genes associated with *M. tuberculosis* virulence (e.g., ESAT-6 like proteins) and pro-apoptotic cascades (Table S2). In summary, our study highlights the importance of combining phenotypic and genotypic assays towards a better understanding of NTM-host “arms race” during infection.

## 5. Conclusions

The major finding of the present work is that phagosome maturation arrest plays a secondary role in intracellular survival of *M. avium* and *M. fortuitum* in comparison to *M. tuberculosis*. Our data suggest that apoptosis mediated by the caspase 8 and caspase 3/7 pathway, but not NO, might play a role in NTM intracellular persistence. Our insights on the genomic traits underlying virulence further emphasized the importance of carrying out multidisciplinary studies in the NTM field, such as simultaneous transcriptomic profiling of both NTM and host cells. 

## Figures and Tables

**Table 1:** NTM persistence in vitro at different pHs.

	Nontuberculous Mycobacteria ID				
pH	*M. smegmatis*	*M. fortuitum*		*M. avium*	
		ATCC 6841	747/08	ATCC 25291	60/08
4.6	-	+	+	+	+
5.4	+	+	+	+	+
6.7	+	+	+	+	+ ^1^

^1^ (+) growth; (-) no growth.

## Supplementary Materials

The following are available online at https://www.mdpi.com/2076-2607/7/5/113/s1, Table S1: Whole-genome sequencing statistics, Table S2: Repertoire of virulence genes in the five NTM genomes, Table S3: Global overview on the differential accessory genome of same-species reference and clinical isolates, Table S4: List of genes likely associated with antibiotic resistance predicted *in silico.*
